# Ileal prolapse through a patent omphalomesenteric duct: a case report

**DOI:** 10.1097/RC9.0000000000000786

**Published:** 2026-08-07

**Authors:** Florent Tshibwid A Zeng, Prince Muteba Katambwa, Chadrack Ndala Mpyana, Ildéphonse Teta Wa Mwanza, Guy-René Ilunga Nday, Sébastien Mbuyi-Musanzayi

**Affiliations:** a Department of Surgery, Faculty of Medicine, Université de Lubumbashi, Lubumbashi, Democratic Republic of the Congo; b Department of Surgery, University Clinics of Medicine, Lubumbashi, Democratic Republic of the Congo; c Department of Anesthesia and Resuscitation, University Clinics of Medicine, Lubumbashi, Democratic Republic of the Congo

**Keywords:** neonate, omphalomesenteric duct remnant, patent vitelline duct, prolapse

## Abstract

**Introduction and clinical importance:**

A patent omphalomesenteric duct is a rare presentation of omphalomesenteric duct remnants. Early diagnosis is essential to avoid subsequent complications.

**Case presentation:**

We report a 14-day-old male patient misdiagnosed as having an iatrogenic injury to the umbilicus. After careful history-taking and a clinical examination that showed a Y-shaped ileal prolapse through the umbilicus and the absence of any signs of trauma, the diagnosis of a patent omphalomesenteric duct complicated by ileal prolapse was made, and the patient was managed in our department with emergency segmental resection of the involved loop. The postoperative course was unremarkable, and the patient was free of symptoms 3 months after surgery.

**Clinical discussion:**

A patent omphalomesenteric duct classically presents as a moist umbilicus with intestinal discharge through the umbilicus. In the absence of experience and a proper physical examination, it can be mistaken for other benign umbilical lesions, such as a granuloma. In such circumstances, a complication may occur, such as ileal prolapse through the patent omphalomesenteric duct.

**Conclusion:**

A patent omphalomesenteric duct is not well recognized among healthcare workers in rural settings, leading to delayed diagnosis and subsequent complications such as ileal prolapse. With appropriate management, outcomes are satisfactory.

## Introduction

During the first 4 weeks of pregnancy, the yolk sac is an important source of nutrients for the embryo through its connection to the midgut via the omphalomesenteric duct (OD). As the placenta becomes fully functional and provides nutrients to the embryo, the yolk sac regresses, with subsequent obliteration of the omphalomesenteric duct from the 5th to the 7th gestational weeks^[^1^]^. Failure of this process leads to OD remnants, which include Meckel’s diverticulum, omphalomesenteric cyst, omphalomesenteric band, umbilical sinus, and patent omphalomesenteric duct (POD)^[^1^]^. The latter is a rare condition, resulting in a congenital complete communication between an ileal loop and the umbilicus. POD occurs in 1 in 15 000–16 000 births^[^2^]^. After umbilical cord separation, POD clinically presents with fecal discharge through the umbilicus^[^1^]^. Due to the rarity of the condition and the lack of specific knowledge of the disease, misdiagnosis and delayed diagnosis are not uncommon and therefore can lead to complications such as ileal prolapse through the POD. Ileal prolapse can result in intestinal obstruction and bowel necrosis from strangulation, similar to intussusception^[^3^]^. We report this case to highlight a rare presentation of POD, which itself is a rare condition, and to show that the misdiagnosis of this condition is still possible in resource-constrained settings.

HIGHLIGHTS

- What is known about the subject: a patent omphalomesenteric duct is one of the rarest types of omphalomesenteric duct anomalies. It usually presents in the neonatal period without complications.
- What this study adds: we report a case of ileal prolapse, a rare complication of a patent omphalomesenteric duct, which was initially misdiagnosed as an iatrogenic injury.

Following the SCARE guidelines^[^4^]^, we report a patient with complicated POD managed at our institution (Supplemental Digital Content 1, available at: https://links.lww.com/IJSCR/A109).

## Case presentation

A 14-day-old male patient was referred to the Department of Surgery of the University Clinics of Lubumbashi for suspected iatrogenic evisceration and bowel injury, with fecal discharge from the umbilicus. Parents reported that the symptoms (a red umbilical mass and fecal discharge from the umbilicus) began 8 hours earlier, following daily umbilical dressings for a moist umbilicus that had been performed since the umbilical cord separated on day 7 of life. The patient was born at full-term to a 24 years-year-old mother after an unmonitored pregnancy. Birth occurred at a medical center via vaginal delivery, with a birth weight of 3400 grams. No specific medical history was reported.

**Figure 1.**
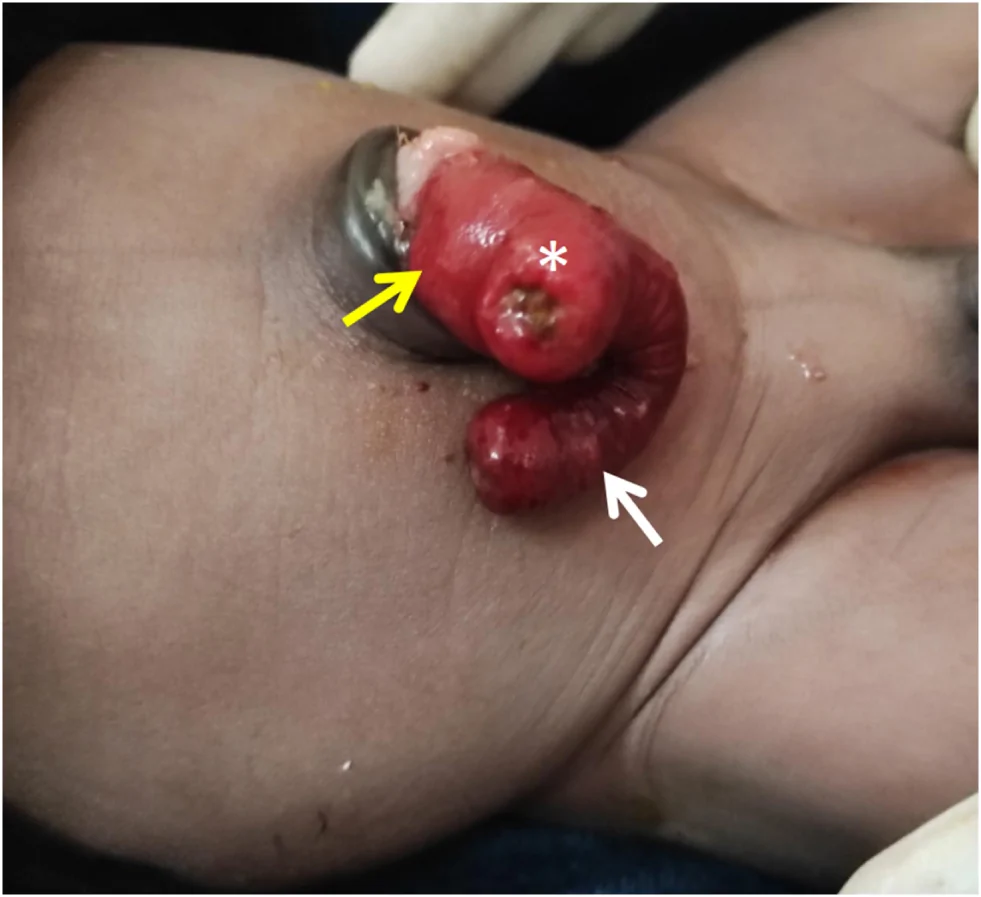
Findings on clinical examination: ileal prolapse through the POD (yellow arrow), with an ischemic proximal segment (white arrow) and a viable distal segment (asterisk).

On admission, the patient had vital signs within the normal range for age. An abdominal dressing with fecal secretions was noted. After removal of the dressing, we noticed a protruding Y-shaped red mass in the umbilical region, with a mucosal appearance, consistent with prolapse of proximal and distal ileum through the umbilicus, associated with passage of feces from the proximal prolapsed segment (Fig. 1). No bleeding from the peri-umbilical skin, nor was any traumatic lesion found, and, because of that, we formally ruled out iatrogenic injury. The remainder of the physical examination was normal. With the typical clinical presentation and experience with previous cases, the diagnosis of ileal prolapse through a patent omphalomesenteric duct was made and emergency surgery was planned, without the need for additional investigation to screen for associated malformations since the patient had no additional symptoms. The patient was kept nil per os, with intravenous hydration, cefotaxime and paracetamol. The full blood count revealed microcytic, hypochromic anemia (10 g/dL). To prevent intraoperative bleeding, a red blood cell transfusion at 15 mL/kg was given 4 hours after admission, with a subsequent increase in hemoglobin to 14 g/dL before surgery.

**Figure 2.**
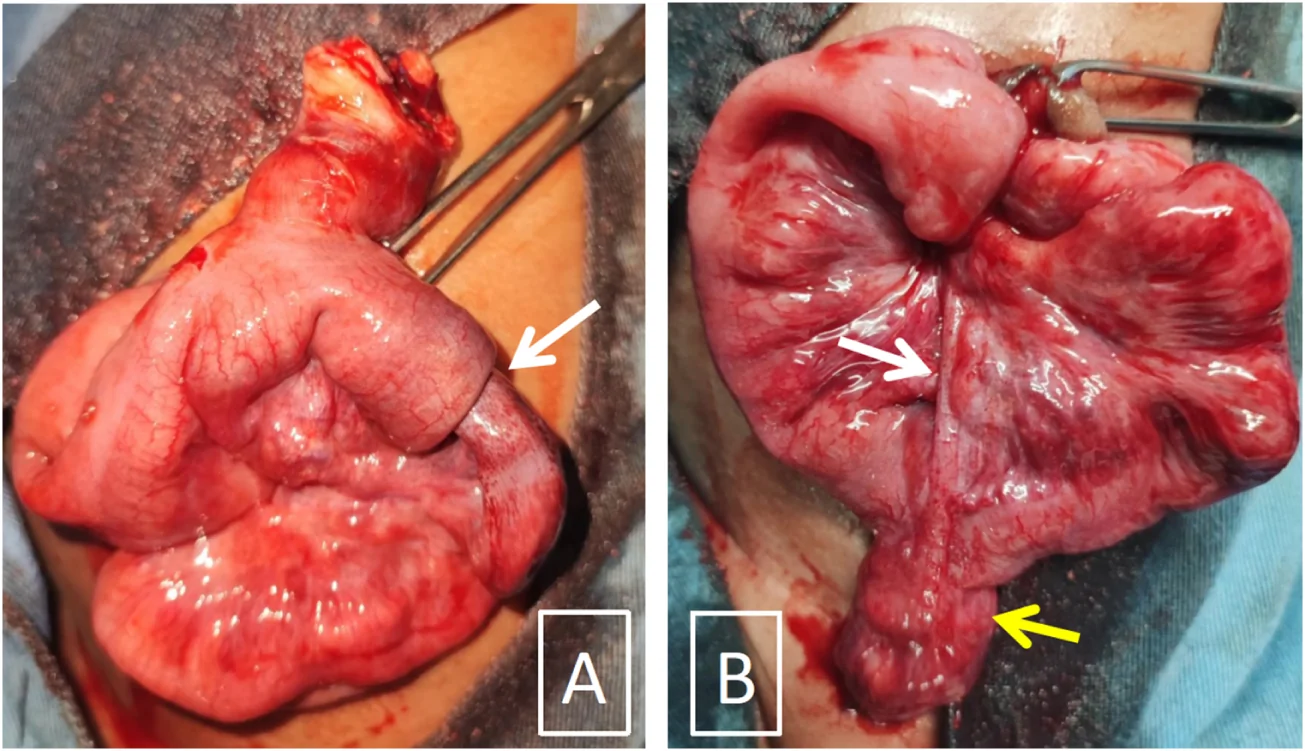
Intraoperative findings. In (A), an intra-abdominal view shows the proximal ileal prolapse through the POD (white arrow), with the distal prolapse already reduced. In (B), the POD is identified (yellow arrow), along with the persistent vitelline artery (white arrow).

**Figure 3.**
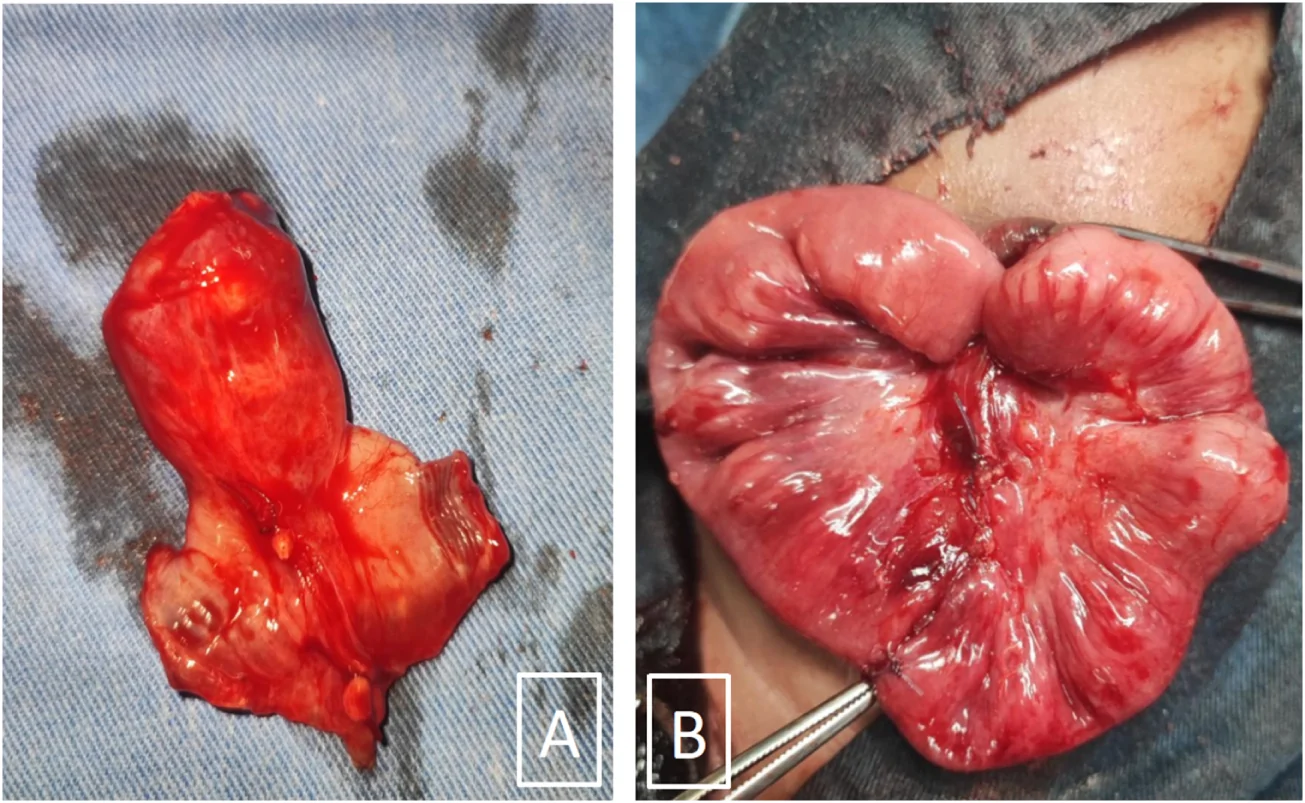
Resection and anastomosis. In (A), the 5-cm resected ileal loop carrying the POD is shown. In (B), the final aspect of the ileal end-to-end anastomosis is shown (forceps).

Surgery was performed through a transumbilical open approach, with ligation of the median umbilical ligament at the lower aspect of the umbilicus and the round ligament at the upper aspect of the umbilicus. After reduction of the ileal prolapse, findings were compatible with the preoperative diagnosis (Fig. 2). We performed a 5-cm segmental ileal resection, removing the carrying loop, followed by an end-to-end anastomosis with continuous sutures using Polyglactin 4/0 (Fig. 3). The umbilical ring was closed with Polyglactin 0 using interrupted sutures. Umbilicoplasty was performed using a purse-string suture with Polyglactin 4/0.

Postoperatively, the patient received maintenance fluids and IV paracetamol. Oral feeding was started 24 hours after surgery, when the patient had resumed bowel movements. On postoperative day 2, the FBC showed resolution of anemia, with a hemoglobin of 16 g/dL. The postoperative course was smooth; the drain was removed on postoperative day 2, and the patient was discharged on postoperative day 5. Pathological analysis of the resected intestinal loop and POD showed a normal ileal lining in both the intestinal loop and the POD, with no ectopic gastric or pancreatic tissue. Six months after surgery, the patient was free of symptoms, with acceptable umbilical cosmesis (Supplemental Digital Content 2, available at: https://links.lww.com/IJSCR/A110). Planned follow-up includes a visit at 1 year postoperatively and then as needed. Due to the risk of adhesive small-bowel obstruction, the parents were instructed to go to the nearest hospital in case of obstructive symptoms.

## Discussion

This study reports a rare case of a patent omphalomesenteric duct complicated by ileal prolapse through the duct, which was initially diagnosed as an umbilical iatrogenic injury. After admission, the 14-day-old patient was managed with a carrying loop resection and anastomosis, with a satisfactory outcome. In Table 1, we subsequently summarized the cases reported in the past 5 years, from 2021 to 2025^[^5–16^]^.

**Table 1:** Summary of recently reported cases of prolapsed POD.

Author, year, country (Reference)	Sex	Age	Intestinal obstruction	Associated anomalies	Imaging investigations	Surgical approach/management of POD	Outcomes
Yigzaw WA, 2025, Ethiopia^[^5^]^	F	4 D	No	Cyanotic CHD, omphalocele, ES, sepsis	Abdominal US, Echocardiogram	Peri-omphalocele/Wedge resection	Death, PoD 2
Hawariat BYW, 2024, Ethiopia^[^6^]^	M	2 Y	No	None	None	Smiley supra-umbilical/Wedge resection	Uneventful, Discharged PoD 4
Cheng F, 2023, China^[^7^]^	F	10 D	No	None	Abdominal CT	Trans-umbilical/Segmental resection	Uneventful, Discharged PoD 7
Martini N, 2023, Syria^[^8^]^	M	17 D	No	None	None	Smiley infra-umbilical/Segmental resection	Uneventful, PoD5
Sandifer SP, 2023, USA^[^9^]^	M	Soon after birth	No	Omphalocele	Echocardiogram, Abdominal US	Trans-omphalocele/Segmental resection	Uneventful, Discharged (PoD NR)
Maulidyan A, 2022, Indonesia^[^10^]^	M	2 D	No	None	Plain x-ray	Median supra-umbilical/Segmental resection	Uneventful, Discharged on PoD 4
Rovillos KP, 2022, Philippines^[^11^]^	F	Soon after birth	No	Omphalocele, ADS, VSD, Respiratory Distress Syndrome, Prematurity	Fistulogram, Echocardiogram	Trans-omphalocele/Fistulectomy	Death, PoD 12
Basu S, 2021, India^[^12^]^	M	1 Mo	Yes	Necrosis of prolapsed ileum	None	Median infra-umbilical/Segmental resection	Uneventful, Discharged (PoD NR)
Gueye D, 2021, Senegal^[^13^]^	F	3 D	No	None	None	Smiley infra-umbilical/Segmental resection	Uneventful, Discharged (PoD NR)
Kumar B, 2021, India^[^14^]^	M	4 Mo	No	None	None	Smiley infra-umbilical/Segmental resection	Uneventful, Discharged PoD 10
Kumar C, 2021, India^[^15^]^	M	47 D	No	Bowel evisceration, Hypokalaemia	None	Trans-umbilical/Segmental resection	Uneventful, Discharged on PoD 7
Moulot MO, 2021, Cote d’Ivoire^[^16^]^	M	3 D	No	Necrosis of prolapsed ileum	None	Trans-umbilical/Segmental resection	Uneventful, Discharged on PoD 3

ASD, atrial septal defect; CHD, congenital heart defect; CT, computed tomography; D, day; ES, Edward’s syndrome; F, female; M, male; Mo, month; NR, not reported; PoD, postoperative day; POD, patent omphalomesenteric duct; US, ultrasound; VSD, ventricular septal defect; Y, year

Patent omphalomesenteric duct (POD) is a rare congenital anomaly, occurring in 1 in 15 000–16 000 births, predominantly in males (77%)^[^2^]^. In POD, prolapse is reported in nearly 41% of cases^[^2^]^. In our review of recent cases of ileal prolapse complicating POD, the male predominance was confirmed, and associated malformations were present in 25% of cases, predominantly omphalocele^[^5,9,11^]^. However, in our patient, no associated congenital malformation was found. When the clinical presentation of prolapse is typical, with a T- or Y-shaped everted intestinal mucosa with or without feces issuing from one side, diagnostic errors are not rare, due to the rarity and poor knowledge of the condition. Authors have reported confusion between ruptured omphalocele and prolapsed POD with associated omphalocele^[^9^]^. In our patient, the initial referral was made on the basis of a suspected iatrogenic injury, which was found to be nonexistent. Among reviewed cases, diagnostic investigations, including fistulogram, abdominal US, and CT scan, were conducted in 33% of cases^[^5,7,9,11^]^. Due to the typical clinical presentation of prolapsed POD and the radiation exposure associated with some investigations (fistulogram and CT scan), we believe these investigations should be reserved for patients with an atypical clinical presentation.

After the diagnosis of prolapsed ileum through POD, emergency management is crucial to prevent bowel necrosis, which was reported in 16% of reviewed cases^[^12,16^]^. After resuscitation, if necessary, different surgical approaches have been reported, with a predominance of three approaches: the smiley infra-umbilical approach^[^8,13,14^]^, the peri-/trans-omphalocele approach^[^5,9,11^]^, and the trans-umbilical approach^[^7,15,16^]^. We favored the latter, as it offers the advantage of access to the abdominal cavity via a scarless surgical procedure. Surgical management of the POD was dominated by segmental resection in 75% of cases^[^7–10,13–16^]^. In fact, due to the possibility of ectopic tissue, fistulectomy or wedge resection of the POD are not warranted. Both may leave residual ectopic tissue, and asymmetry after wedge resection may result in a lead-point for intussusception^[^17^]^. As performed in our patient, segmental resection is the method of choice, with extensive ileal resection in cases of bowel necrosis^[^3,18^]^. The postoperative course after surgery for POD with ileal prolapse is reported to be smooth, but mortality can occur. Among reviewed cases, mortality occurred in 2 patients (16.7%), due to concurrent complications: a cyanotic congenital heart defect in one patient and respiratory distress syndrome in another^[^5,11^]^.

## Strengths and limitations

This study reports on a patient with a rare complication of a patent omphalomesenteric duct, itself a rare presentation of omphalomesenteric duct anomalies. The main limitation is the lack of microscopic images, which would enrich the iconography of this work.

## Conclusion

The patent omphalomesenteric duct is poorly recognized by healthcare workers in rural areas and may be mistaken for an iatrogenic injury following umbilical cord separation. Delayed diagnosis may result in complications, such as ileal prolapse through the patent duct. When such a complication is promptly managed, outcomes are satisfactory.

## Data Availability

The data that support the findings of this study are available from the corresponding author upon reasonable request.

## Patient perspective

The patient’s parents were satisfied with the timely and successful management of their child’s condition using “scarless” surgery and with acceptable umbilical cosmesis.
